# Pre-disaster social support is protective for onset of post-disaster depression: Prospective study from the Great East Japan Earthquake & Tsunami

**DOI:** 10.1038/s41598-019-55953-7

**Published:** 2019-12-19

**Authors:** Yuri Sasaki, Jun Aida, Taishi Tsuji, Shihoko Koyama, Toru Tsuboya, Tami Saito, Katsunori Kondo, Ichiro Kawachi

**Affiliations:** 1 Department of International Health and Collaboration, National Institute of Public Health, Wakō, Japan; 2 Department of International and Community Oral Health, Tohoku University Graduate School of Dentistry, Sendai, Japan; 3 Department of Social Preventive Medical Sciences, Centre for Preventive Medical Sciences, Chiba University, Chiba, Japan; 4 Department of Cancer Epidemiology, Cancer Control Centre, Osaka International Cancer Institute Japan, Osaka, Japan; 5 Department of Social Science, Centre for Gerontology and Social Science, National Centre for Geriatrics and Gerontology, Ōbu, Japan; 6 Department of Gerontological Evaluation, Centre for Gerontology and Social Science, National Centre for Geriatrics and Gerontology, Ōbu, Japan; 7 Centre for Well-Being and Society, Nihon Fukushi University, Nagoya, Japan; 8 Department of Social and Behavioral Sciences, Harvard School of Public Health, Boston, MA United States of America

**Keywords:** Health care, Epidemiology

## Abstract

We examined whether pre-disaster social support functions as a disaster preparedness resource to mitigate post-disaster depressive symptoms among older survivors of the 2011 Great East Japan earthquake and tsunami. The participants were 3,567 individuals aged ≥65 years living in Iwanuma city who completed a baseline survey as part of the nationwide Japan Gerontological Evaluation Study seven months before the disaster. A follow-up survey was administered approximately 2.5 years after the disaster. The analysis included a total of 2,293 participants, and social support (giving and receiving emotional & instrumental help) before the disaster was measured using four items. Depressive symptoms were assessed using the GDS with a cut-off score of 4/5 (not depressed/depressed). We discovered that participants who gave and received emotional and instrumental support before the disaster were significantly less likely to develop depressive symptoms after the disaster compared to those without support (ARR = 0.70; 95% CI: 0.56–0.88). The risk of the onset of depressive symptoms was 1.34 (95% CI: 1.03–1.74) among those who experienced disaster damages but had also given and received social support, and 1.70 (95% CI: 1.03–2.76) among those who experienced damages but lacked support. Strengthening social aid may help cultivate psychological resilience to disasters.

## Introduction

Natural disasters, such as earthquakes and tsunamis, have wide-ranging impacts on psychological functioning. The prevalence of post-traumatic stress disorder (PTSD) and depressive symptoms noticeably increases after disasters^1–3^. Disaster preparedness—that is, the necessary knowledge, capabilities, and actions taken to reduce the harm caused by a disaster—is recognised as a key issue for disaster resilience, as it can reduce disaster damage and promote recovery. A review of the literature on emergency preparedness^4^ confirmed that concrete action by governments and local authorities (such as the distribution of informational booklets about disaster preparedness among households, as well as encouraging disaster preparedness drills/exercises by voluntary associations) can be effective in promoting it^5,6^. By contrast, individual characteristics associated with lower disaster preparedness include older age, physical disability, lower educational attainment, and lower household income^7^. Other studies point to community characteristics that favour disaster preparedness, such as the presence of strong social and economic infrastructure, as well as strong social cohesion and shared values^4,8–10^. However, the exact resources that mitigate the adverse health effects of a disaster, or directly improve health after a disaster, are unclear. Determining such resources and quantifying their contribution to psychological health is necessary for appropriate disaster preparedness planning.

Social support is considered as disaster preparedness resource^11,12^; it is robustly linked to improved physical and psychological health in the general population^13–16^. In the aftermath of disasters, social support has been linked to better mental health outcomes for survivors^11,12,17–21^. However, previous studies have been limited by their cross-sectional design, i.e. social support is assessed in the post-disaster situation so that it is not possible to rule out reverse causality (for example, survivors with more problems receive more support.)^17–21^

In addition, there is a debate as to whether social support promotes health via a main effect or a stress-buffering effect. According to the stress-buffering hypothesis, social support is protective in the presence of stress or trauma, whereas the main effect hypothesis posits that social support promotes mental health regardless of the experience of stress or trauma^13^. It can be difficult to distinguish between these two models because of selection bias—people suffering from stressful events, for instance, tend to seek out social support. Most existing studies have not been able to address the endogeneity of the stress event. However, because natural disasters are a type of exogenous shock, examining the effects of pre-disaster social support on mental health enables us to tease out the two mechanisms. Taking advantage of a unique ‘natural experiment’^22^ associated with the 2011 Great East Japan earthquake and tsunami, we investigated whether pre-disaster social support functions as a disaster preparedness resource for the onset of post-disaster depressive symptoms.

## Results

Among the 2,242 participants included in the analysis, the average age was 72.8 ± 5.8 years at baseline, and 53.7% of them were women (Table 1). At the follow-up survey, 363 participants had developed depressive symptoms (cumulative incidence = 16.2%). The mean weighted support scores tended to be higher among individuals who did not develop depressive symptoms at follow-up than among those who did (score: 3.8 vs. 3.6, p < 0.01) (Table 1). Furthermore, individuals who experienced housing damage and loss of close relative(s) were more likely to develop depressive symptoms compared to those who did not (18.3% vs 13.2%, p < 0.01; 19.7% vs 14.9%, p < 0.01) (Table 1).

**Table 1:** Socio-demographic characteristics of study participants (n = 2,242).

		Total		GDS score at follow-up in 2013				p-value
				Not depressed (GDS score <5)		Depressed (GDS score ≥5)		
		n = 2,242		n = 1,879	83.8%	n = 363	16.2%	
Giving emotional social support	Yes	2,031		1,712	84.3%	319	15.7%	<0.01^a^
	No	134		101	75.4%	33	24.6%	
	Missing	77		66	85.7%	11	14.3%	
Giving instrumental social support	Yes	1,935		1,646	85.1%	289	14.9%	<0.01^a^
	No	198		146	73.7%	52	26.2%	
	Missing	109		87	79.8%	22	20.2%	
Receiving emotional social support	Yes	2,044		1,720	84.2%	324	15.1%	0.11^a^
	No	127		100	78.7%	27	21.3%	
	Missing	71		59	83.1%	12	16.9%	
Receiving instrumental social support	Yes	2,135		1,796	84.1%	339	15.9%	<0.05^a^
	No	68		51	75.0%	17	25.0%	
	Missing	39		32	82.1%	7	17.9%	
Social support score	Mean (±SD)	3.8	(0.7)	3.8	(0.6)	3.6	(0.8)	<0.01^b^
Age	Mean (±SD)	72.8	(5.8)	72.6	(5.7)	74.1	(6.2)	<0.01^b^
Gender	Male	1,039		876	84.3%	163	15.7%	0.38^a^
	Female	1,203		1,003	83.4%	200	16.6%	
Equivalized income	High	484		461	95.2%	23	4.8%	<0.01^a^
	Middle	869		752	86.5%	117	13.5%	
	Low	812		666	82.0%	146	18.0%	
	Missing	77		0	0.0%	77	100.0%	
Living status	Not alone	2,039		1,717	84.2%	322	15.8%	<0.01^a^
	Alone	158		126	79.7%	32	20.3%	
	Missing	45		36	80.0%	9	20.0%	
GDS score at 2010	Mean (±SD)	1.6	(1.3)	1.5	(1.3)	2.4	(1.3)	<0.01^b^
Housing damage	No damage	931		808	86.8%	123	13.2%	<0.01^a^
	Some damage	1,268		1,036	81.7%	232	18.3%	
	Missing	43		35	81.4%	8	18.6%	
Bereavement	No loss of close relative(s)	1,639		1,395	85.1%	244	14.9%	<0.01^a^
	Loss of close relative(s)	603		484	80.3%	119	19.7%	
	No loss of close friend(s)	1,880		1,578	83.9%	302	16.1%	0.92^a^
	Loss of close friend(s)	362		301	83.1%	61	16.9%	

Participants with limitations performing activities of daily living (ADL) (i.e., independent walking, bathing, and, toileting) and participants receiving public long-term care insurance benefits were excluded.

Participants who had mild or more severe depression (the Geriatrics Depression Scale (GDS) score ≥5) in the baseline survey of 2010 were also excluded.

Weighted social support score was calculated by using a polychoric correlation matrix in a factor analysis model.

SD: Standard Deviation.

a: p-value for chi-square test; b:p-value for t-test.

Table 2 shows the results of the Poisson regression analysis with a robust error variance in the association between having social support before the disaster and the onset of depressive symptoms in the dataset with multiple imputation (MI) (n = 2,293). The point estimates for the adjusted model show protective influence on depressive symptoms for both giving emotional and/or instrumental social support, and receiving social support [adjusted rate ratio (ARR) = 0.63, 95% confidence interval (CI) = 0.39–1.00; ARR = 0.68, 95% CI = 0.35–1.30, respectively]. The individuals with all four aspects (i.e. giving and receiving emotional & instrumental supports) were significantly less likely to report depressive symptoms than the others, even after adjusting the covariates of demographic variables (i.e. age, sex, living status, equivalized income) and disaster damages [i.e. housing damage, loss of close relative(s), and loss of close friend(s)] (ARR = 0.70, 95% CI = 0.56–0.88), indicating that social support had significant influence (Table 2).

**Table 2:** Multivariate Poisson regression (A)RR and 95% CIs from MI analysis for support factors of depressive symptoms (n = 2,293).

Social support	Crude Model A1				Adjusted Model A2			
	RR		95% CI		ARR		95% CI	
**Giving social support**								
Emotional and/or Instrumental social support	0.54	*	0.34	0.86	0.63		0.39	1.00
No emotional and no instrumental social support	1.00				1.00			
**Receiving social support**								
Emotional and/or instrumental social support	0.63		0.34	1.16	0.68		0.35	1.30
No emotional and no instrumental social support	1.00				1.00			
**Giving and receiving instrumental & emotional support**								
Four social supports	0.63	**	0.51	0.79	0.70	**	0.56	0.88
Everybody else	1.00				1.00			

MI: Multiple Imputation, RR: Rate Ratio, ARR: Adjusted Rate Ratio, CI: Confidence Interval. Model A2 adjusted for age, sex, living status (alone or not alone), equivalized income, all types of disaster damage [housing damage, loss of close relative(s), and loss of close friend(s)]. Four social supports: Giving and receiving emotional & instrumental support *P-value for < 0.05; **P-value for < 0.01.

**Figure 1:**
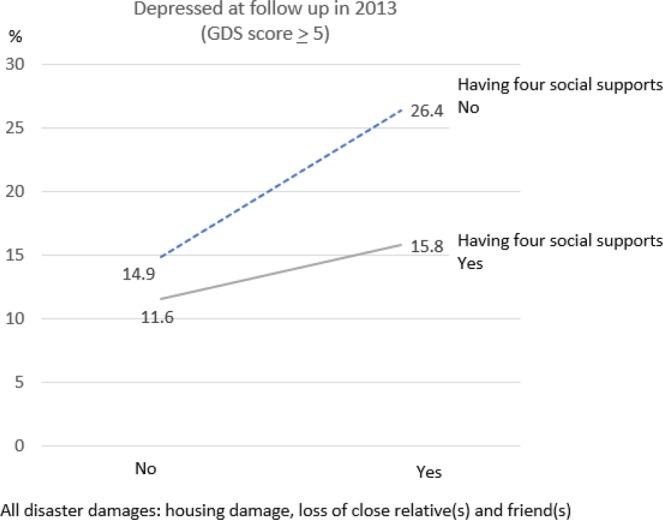
Incidence of depressive symptoms among those who had disaster damages and who did not according to the supports which they gave and received. *Four social supports: Giving and receiving instrumental & emotional support.

To test the buffering effect of social support, Table 3 & Fig. (1) show the onset of depressive symptoms according to disaster damage and social support. Those who reported experiencing disaster damage [housing damage, loss of close relative(s) and friend(s)] tended to have a higher incidence of depressive symptoms. This trend was more pronounced among survivors who lacked social support. In stratified analysis, the risk of the onset of depressive symptoms was 1.34 (95% CI: 1.03–1.74) among those who experienced disaster damage but also gave and received social support, while it was 1.70 (95% CI: 1.03–2.76) among those who experienced damage but lacked support (Table 3). However, in the pooled analysis, the interaction between social support and disaster damage was not formally statistically significant (ARR = 0.77; 95% CI: 0.43–1.35).

**Table 3:** Multivariate Poisson regression (A)RR and 95% CIs from MI analysis for disaster damage factors of depressive symptoms.

All types of disaster damage	Crude Model A1				Adjusted Model A2			
	RR		95% CI		ARR		95% CI	
**Among**								
those who had four social supports (n = 1,903)	1.30		0.997	1.70	1.34	*	1.03	1.74
other than those above (n = 376)	1.69	*	1.03	2.77	1.70	*	1.03	2.76

MI: Multiple Imputation, RR: Rate Ratio, ARR: Adjusted Rate Ratio, CI: Confidence Interval. Model A2 adjusted for age, sex, living status (alone or not alone), equivalized income. All types of disaster damage: housing damage, loss of close relative(s) and friend(s). Four social supports: Giving and receiving emotional & instrumental support *P-value for < 0.05.

As a sensitivity analysis, we analysed five categories of social support instead of the binarized version: 0 (reference), one form of social support, two forms of social support, three forms of social support, and four forms of social support. The variable for disaster damage was also treated as a continuous variable, and we included individuals whose GDS score at baseline was five or above. The result of this sensitivity analysis yielded the same conclusion as the main analysis, i.e., participants with higher social support had lower risk of the onset of depressive symptoms (Appendix 1).

## Discussion

The main contribution of this study is having reported the result of a natural experimental to test whether *pre*-disaster social support functions as a disaster preparedness resource. A total of 16.2% of older survivors developed depressive symptoms after the disaster. Survivors who had all four types of social support (giving and receiving emotional & instrumental support) prior to the disaster experienced a lower risk of developing depressive symptoms compared to those who did not have such support, even after adjusting for demographic and disaster-damage-related variables. Moreover, the risk of the onset of depressive symptoms tended to be higher among those who experienced disaster damage and did not give and/or receive social support compared to those who had social support. However, the interaction between social support and disaster damage was not statistically significant in the multivariate analysis.

The results of a past meta-analysis indicated that the incident depressive symptoms after a natural disaster ranged from 5.8% to 54%^23^; the 16.2% we found falls within this range, although we cannot directly compare the incidence in this study with that of previous studies because of differences in the study designs, the time after disasters, the measure of depressive symptoms used, and the target population. To directly compare the incident depressive symptoms among non-disaster exposed population, we calculated the incidence from the participants of the Japan Gerontological Evaluation Study (JAGES) not living in Iwanuma city, where the current study focused; the incidence was 11.7% (3,597 case among 30,763 participants), relatively lower compared to that of participants in Iwanuma city.

Although marginally significant, both giving and receiving emotional & instrumental support appear to have an influence on the onset of depressive symptoms (Table 2). The majority of the previous studies suggested that emotional support is a constant predictor of health and wellbeing in the general population^24–27^, and a review article concluded that the strongest and most consistent findings were protective effects of perceived emotional support and instrumental support regarding depression in the general population^28^. Although this study was a prospective cohort study and we were able to target members of the population who were not depressed at baseline, the results in this study were in accord with the previous ones. One possible explanation for the similarity might be a characteristic of the post-disaster context (i.e. after a major disaster—when there is widespread destruction of property and loss of wealth). The survivors may be more likely to benefit from both forms of social support, since the support providers may be more likely to have empathy for the population affected by disaster than for the rest^29^. There is also a possibility that survivors themselves providing social support to others might increase their motivation to live. It may suggest that emotional and instrumental social support track each other and boost the protective influence on the onset of depressive symptoms in the disaster-affected area.

Rather few studies have examined the impact of pre-disaster social support on mental health using a prospective study design^11,12^. Still, their results were consistent with ours. One previous study among low-income mothers did find that higher pre-disaster perceived social support would be predictive of lower pre-disaster psychological distress, lower hurricane-related stressors, and higher post-disaster perceived social support; these variables would, in turn, predict lower post-disaster psychological distress^11^. Another study also indicated that pre-existing deficits in social resources might indirectly affect longer-term post-traumatic stress and general psychological distress by increasing risk for disaster-related stressors among low-income mothers^12^. It suggests that pre-disaster social support can aid in disaster preparedness among low-income mothers. Our study, however, was focused on the other vulnerable population in more unpredictable natural disasters (i.e. earthquakes and tsunamis) than those economically poor in more predictable ones (i.e. hurricanes).

We endorse a number of possible explanations for the association between pre-disaster social support and the onset of depressive symptoms after the disaster in our study. First, having had social support before the disaster suggests that individuals might have been more prepared to help (as well as receive help from) family, friends, and neighbours in emergencies, which could have lowered the risk of depressive symptoms^30^. Second, having social support is related to altruistic behaviour, which could have helped reduce the participants’ emotional stress during the disaster as well as helped them develop a mindset and perspective conductive to psychological toughness. This possibility is supported by the positive association between social support and psychological toughness^11,31,32^. Third, biological mechanisms, especially inflammatory processes, might partially explain the results. Although research on the relationship between social support and biological mechanisms (i.e., inflammation) has obtained inconsistent findings^33^, a review article did show that psychosocial factors might more readily affect immune functions in older individuals: older adults often show greater immunological impairment to stress than younger adults^34^. Fourth, participants with greater social support might have a more optimistic personality than those with less social support. Optimism is defined as a disposition or tendency to look on the more favourable side of events or conditions and to expect the most favourable outcome^35^. This trait is believed to be a personality trait that helps people cope with the negative effects of stress^36,37^, and promote better emotional adjustment and physical health^38^. People who rate themselves as high in optimism also tend to report higher wellbeing than those who rate their optimism as low do, regardless of their perceived stress^37^. Moreover, optimistic individuals tend to report greater satisfaction with support^39^, as well as less loneliness and greater feelings of support in general. For example, optimistic women tend to perceive higher levels of support following breast cancer surgery^40^. Accordingly, how perception of social support influences depressive symptoms after a disaster might be a function of personality traits such as optimism. These four might be the possible reasons for suppressing the onset of depressive symptoms.

This study has various implications. The most obvious is that social support in daily life might help older adults maintain their psychological health following disasters; in other words, social support might function as a resource for disaster preparedness regardless of the size of the damages. Public health interventions can therefore increase older people’s opportunities to participate in social activities and improve their social interactions in daily life^41^.

Moreover, the influence of social support on the onset of depressive symptoms appears to be similar whether the social support is received or provided. As a result, it is important for older people to have not only people who can care for them when they are sick, but also opportunities to adopt that role themselves. Future research should explore not only the quantity of support offered, but also the quality of the support, as this could help in explaining the mechanism by which support influences the onset of depressive symptoms in natural disasters.

**Figure 2:**
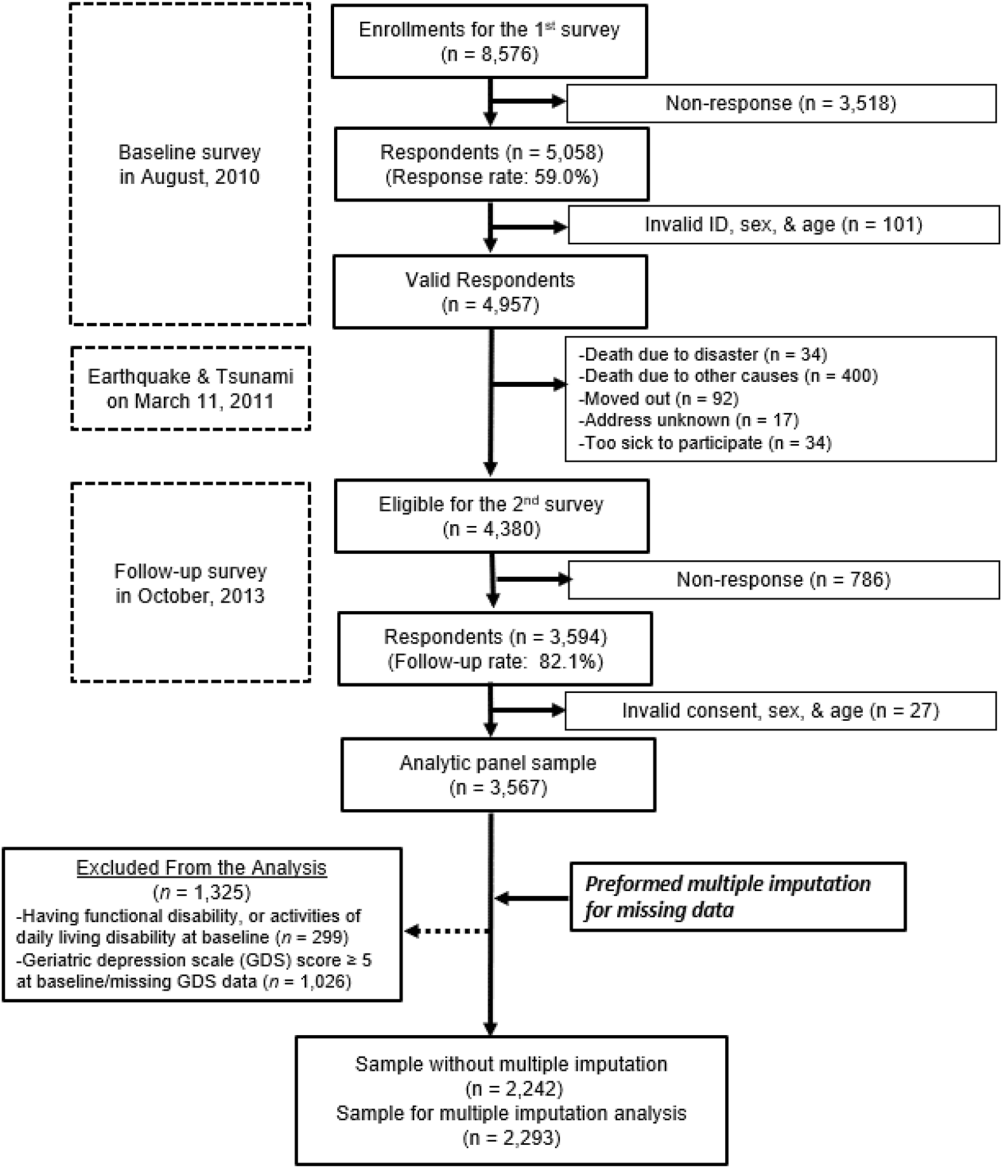
Participant flow of the present study.

Our results, however, should be interpreted with some caution. Since the measure of presence of depressive symptoms relied on participants’ recalling of the events, which is a common issue for studies relying on self-reports, the results do not necessarily translate to clinical significance. However, GDS is an instrument for screening major depression, as it sets the cut-off value and examines the differences between those who are at an increased risk of onset of major depression and those who are not after a disaster; this is the original significance of this study. Additionally, the mitigating impact on the onset of depressive symptoms (ARR = 0.70) due to receiving all types of social support was comparable to the aggravating impact of experiencing all types of disaster damage (ARR = 1.42, data not shown). There is also a possibility that individuals whose depressive symptoms increased during the time between the two surveys were also more likely to recall personal experiences of disaster damages selectively^42^. Our use of a four-item scale to assess the exchange of social support was relatively crude, and likely to be prone to measurement error. Nonetheless, the items were previously developed and validated to measure the giving and receiving of emotional and instrumental support^43^. In addition, there is a possibility that those who provided support to others might have lost their lives on the day of the disaster, as a result of helping others^44^. However, the overall number of participants who lost their lives on the day of the disaster was fortunately low (n = 34)—see Fig. (2) flowchart; hence, we believe that the impact on our analyses was small. On the other hand, male survivors and those living alone were at high risk of social isolation in Japan^45^, and may have found it difficult to give and receive social support. Although our analyses controlled for these variables, further analyses are needed to determine the impact of both social isolation and social support on the health of disaster survivors. The generalisability of the results is also limited to healthy older adults who responded to both the baseline and follow-up surveys. Excluding older people who dropped out after the baseline survey, had GDS scores of five or more, ADL impairments, and received long-term care insurance benefits at baseline, the present analyses may have led to the underrepresentation of the onset of depressive symptoms. For example, the GDS scores at 2010 and 2013 were both higher among those who were excluded than those were included in the analysis (Appendix 2). Therefore, we might have underrepresented participants more vulnerable to depressive symptoms. Furthermore, cultural differences in seeking and using social support should be considered. A study on culture and social support found that Asians and Asian-Americans psychologically benefit from implicit social support (focusing on valued social networks including instrumental aid) to a greater extent than from explicit social support (seeking and using advice and emotional solace); the reverse was true for European-Americans^46^. That is, the influence of social support may vary depending on the cultural emphasis on maintaining harmonious social relationships on the one hand, and an emphasis on self-expression and verbal sharing of thoughts and feelings (as in western settings) on the other^46,47^.

Although our focus was on whether emotional/instrumental types of social support (giving vs. receiving) are associated with the onset of depressive symptoms after a natural disaster, it is also important to know the source/target of social support. Appendix 3 suggests that spouses matter most for receiving emotional & giving instrumental social support, while friends matter most for giving emotional social support. For social support after the disaster, however, spouses matter for all four types of social support, while friends matter most for giving emotional social support, in the same measure as before the disaster. In addition, children and relatives matter most for giving instrumental social support after the disaster.

The present study also had several strengths. Since social support was assessed seven months before the disaster, there is no possibility of recall bias (i.e. traumatic experiencing leading to biased recalling). It is quite likely that people’s pattern of social support changed after the disaster due to traumatic experiences. However, the primary focus of our study was to examine whether social support can act as a pre-disaster resource for resilience. In addition, because of Japan’s compulsory system of domiciliary registration, which requires all residents to notify authorities of address changes, the number of individuals who dropped out at follow-up in our dataset was quite low (11.6%); consequently, the degree of bias induced by loss to follow-up was likely small^48^. We also used the MI procedure to minimize the impact of missing data as far as possible.

In conclusion, older survivors with social support before the Great East Japan Earthquake and Tsunami had a lower risk of developing depressive symptoms after the disaster compared to those who did not have such support; on the other hand, those who reported experiencing disaster damage and lacking support tended to have a higher incidence of depressive symptoms. As natural disasters become more frequent, it is increasingly important to monitor not only the psychological health of survivors in the aftermath, but also the individuals’ level of contact or connection with others prior to the disaster as a form of preparedness for emergencies. It is necessary to further investigate social support as a form of disaster preparedness and how to utilize it to foster survivors’ resilience following a disaster.

## Methods

### Study design and participants

This study was a part of the Japan Gerontological Evaluation Study (JAGES), which began in 2010 as a nationwide, population-based, prospective cohort study investigating the predictors of physical and psychological health in community-dwelling Japanese older adults^42,49,50^. In the present longitudinal study, we used panel data from two waves of the JAGES survey.

One of the original field sites of the JAGES cohort was Iwanuma city, Miyagi Prefecture, which is located roughly 80 km west of the epicentre of the 2011 Great East Japan Earthquake. Iwanuma city (total population: approx. 44,000) suffered tremendous damage from the Great East Japan Earthquake and Tsunami, with 180 people being killed^51^ and 48% (29 km^2^) of the land becoming inundated by seawater^52^.

Surveys were mailed to all residents of Iwanuma city aged 65 years or older in August 2010 (i.e. seven months before the disaster) and again 2.5 years after the disaster, in October 2013, since JAGES surveys have been conducted almost every three years. The response rate to the baseline survey was 59.0% (n = 5,058). Of these, 34 people died on the day of the disaster, and an additional 400 people died of natural causes before the follow-up survey. After excluding individuals who moved out of the area (n = 92), who were lost to follow-up because they lacked a known forwarding address (n = 17), or were too sick to participate again (n = 34), 4,380 people were considered eligible for the second survey. Of these, 3,594 people responded to it (response rate = 82.1%). After excluding participants with invalid consent forms, a total of 3,567 older adults were found to have participated in both surveys (participation rate = 81.4%)^42^ (Fig. 2).

We excluded participants who met the following criteria: those whose GDS score was five or greater at baseline; those who reported limitations in their activities of daily living at baseline (i.e. showed dependence for walking, bathing, and going to the toilet); finally, those who received public long-term care insurance benefits. Further details of the Iwanuma study can be found elsewhere^48,53,54^. We used data from the remaining 2,242 participants (1,039 male and 1,203 female), and 2,293 participants were analysed after MI.

### Outcome variable: onset of depressive symptoms following a disaster

Our primary outcome was the onset of depressive symptoms measured by the 15-item Geriatric Depression Scale (GDS), which has been previously validated^55,56^. Using the 15-item GDS, we defined the onset of depressive symptoms as having a score of less than five in 2010 and a score of five or more in 2013^54,57^. The GDS includes self-reported yes/no questions and scored as either zero or one point in each. It has well-established validity and reliability for assessing depressive symptoms in older adult populations^53,55,56,58^. In this study, we used a score of four or higher as the cut-off point since it has a sensitivity of 0.96 and specificity of 0.95 for predicting depression in the Asian context^56^.

### Predictor variable: social support

Social support (including emotional and instrumental support) was assessed by asking the following four questions: ‘Do you listen to someone else’s concerns and complaints?’ (giving emotional social support); ‘Do you take care of someone who is sick?’ (giving instrumental social support); ‘Do you have someone who listens to your concerns and complaints?’ (receiving emotional social support); ‘Do you have someone who takes care of you when you are sick?’ (receiving instrumental social support). For all these questions, the possible responses were (1) none (2) spouse, (3) children living together, (4) children or relatives living apart, (5) neighbour, (6) friend, and (7) other. Based on their responses, participants were categorised as ‘having no social support’ (i.e. answering with ‘none’) or ‘having social support’ (answering with any of choices 2 to 7).

### Covariates: demographic characteristics and disaster damage

Demographic characteristics at baseline (sex, age, equivalised income, living status) and disaster damage [housing damage, and losing close relative(s) and friend(s) in the disaster] were adjusted in the multivariate model. These variables were selected based on the data from previous studies in which the outcomes were depressive symptoms on similar populations^48,54^. Information on sex and age was obtained from the government register in 2010. Equivalised household income and living status were obtained using data from the self-report questionnaire. Household income was equivalised in order to adjust for differences in household size (i.e. to correct for the fact that two households with the same income could have different standards of living depending on the number of people living in it). We used the standard procedure of dividing the gross household income by the square root of the number of people in the household^59^. It was then divided into three categories in terms of Japanese yen (2017 average exchange rate: 112 yen = 1 US dollar): low (less than 2 million yen), middle (2–3.99 million yen), and high (4 million yen and over), considering the criteria used in a national survey in Japan^60^.

Based on a previous study^54^, housing damage was assessed by asking each respondent about the extent of the property damage they experienced. Possible answers were ‘not affected’, ‘minor damage’, ‘major damage’, and ‘total collapse’. These categories were based on individual inspection by two assessors from the municipality in order to determine government compensation. From these responses, we formulated two categories: damage or no damage. Loss of relationships due to the disaster was evaluated via the question ‘did you lose a close relative(s) or friend(s) in the earthquake?’, for which participants could provide multiple possible answers. Their answers were categorised as follows: losing close relative(s) or not, and losing close friend(s) or not.

### Statistical analysis

The relationship between the onset of depressive symptoms and social support was examined using a Poisson regression analysis with a robust error variance because the cumulative incidence of depressive symptoms was over 10% during the follow-up period in the cohort. This means that it was possible for the odds ratio obtained via ordinary logistic regression analysis to overestimate the risk^61,62^. The results are presented as ARRs with 95% CI. In the final model, age, sex, equivalised income, living status, and disaster damages were adjusted (Table 2). We also examined the effect of disaster damages on the onset of the depressive symptoms stratified by social support (Table 3). Multiple imputation by chained equations was applied to missing responses. We produced 20 imputed datasets, and analyses were conducted for each of the 20 imputed datasets. Using Rubin’s rule, single mean estimates and adjusted standard errors were obtained. STATA version 14 (Stata Corp, College Station, TX) was used for all analyses, and the statistical significance level set at *P* < 0.05.

### Ethical considerations

The survey protocol was approved by the human subjects’ committee of the Harvard T.H. Chan School of Public Health as well as the human subjects’ committees of Tohoku University, Nihon Fukushi University, and Chiba University. Informed consent was obtained from all participants at the time of data collection. Voluntary participation and right to withdraw at any time were assured. This study conformed to the principles of the Declaration of Helsinki.

## Supplementary information

Appendix 1, Appendix 2, Appendix 3

## Data Availability

All data associated with this study have been included in the manuscript.
