# Supplementary material for: Pre-disaster social support is protective for onset of post-disaster depression: Prospective study from the Great East Japan Earthquake & Tsunami
Source: Sci Rep. 2019 Dec 19;9:19427. doi: 10.1038/s41598-019-55953-7 (PMC6923367; doi:10.1038/s41598-019-55953-7)
Supplement: Supplementary file 1 — Appendix 1, Appendix 2, Appendix 3 [file 41598_2019_55953_MOESM1_ESM.pdf]

Pre-disaster social support is protective for onset of post-disaster depression: Prospective study from the Great East Japan Earthquake & Tsunami

Yuri Sasaki[1*], Jun Aida[2], Taishi Tsuji[3], Shihoko Koyama[4], Toru Tsuboya[2], Tami Saito[5], Katsunori Kondo[3,6,7], and Ichiro Kawachi[8]

[1]Department of International Health and Collaboration, National Institute of Public Health, Japan

[2]Department of International and Community Oral Health, Tohoku University Graduate School of Dentistry, Japan

[3]Department of Social Preventive Medical Sciences, Centre for Preventive Medical Sciences, Chiba University, Japan

[4]Department of Cancer Epidemiology, Cancer Control Centre, Osaka International Cancer Institute Japan

[5]Department of Social Science, Centre for Gerontology and Social Science, National Centre for Geriatrics and Gerontology, Japan

[6]Department of Gerontological Evaluation, Centre for Gerontology and Social Science, National Centre for Geriatrics and Gerontology, Japan

[7]Centre for Well-Being and Society, Nihon Fukushi University, Japan

[8]Department of Social and Behavioral Sciences, Harvard School of Public Health, Boston, MA, United States of America

Contact Information for all authors

Correspondence

Yuri Sasaki, Department of International Health and Collaboration, National Institute of Public Health, Japan, 2-3-6 Minami, Wako-shi, Saitama 351-0197, Japan

Email: sasakiy1006@gmail.com; Phone: +81-48-458-6149; Fax:+81-48-469-2768

Jun Aida, Department of International and Community Oral Health, Tohoku University Graduate School of Dentistry, Japan, 4-1 Seiryo-cho, Aoba-ku, Sendai-shi, Miyagi 980-8575, Japan

Email: aidajun@m.tohoku.ac.jp; Phone: +81-22-717-7639; Fax: +81-22-717-7644

Taishi Tsuji, Department of Social Preventive Medical Sciences, Center for Preventive Medical Sciences, Chiba University, Japan, 1-8-1 Inohana, Chuo-ku, Chiba-shi, Chiba 260-8670, Japan
Email: tsuji.t@chiba-u.jp; Phone:+81-43-226-2803; Fax:+81-43-226-2018

Shihoko Koyama, Department of Cancer Epidemiology, Cancer Control Center, Osaka International Cancer Institute Japan
3-1-69 Ohtemae, Chuo-ku, Osaka 541-8567, Japan
Email: shihoko-koyama@umin.ac.jp; Phone: +81-6-6945-1181

Toru Tsuboya, Department of International and Community Oral Health, Tohoku University Graduate School of Dentistry, Japan, 4-1 Seiryo-cho, Aoba-ku, Sendai-shi, Miyagi 980-8575, Japan
Email: tsubo828@med.tohoku.ac.jp; Phone:+81-22-717-7639; Fax:+81-22-717-7644

Tami Saito, Department of Social Science, Center for Gerontology and Social Science, National Center for Geriatrics and Gerontology, Japan, 7-430 Morioka-cho, Obu-shi, Aichi 474-8511, Japan
Email: t-saito@ncgg.go.jp; Phone:+81-56-246-2311; Fax:+81-56-244-6539

Katsunori Kondo, Department of Social Preventive Medical Sciences, Center for Preventive Medical Sciences, Chiba University, Japan, Department of Gerontological Evaluation, Center for Gerontology and Social Science, National Center for Geriatrics and Gerontology, Japan & Center for Well-Being and Society, Nihon Fukushi University, Japan, 1-8-1 Inohana, Chuo-ku, Chiba-shi, Chiba 260-8670, Japan
Email: kkondo@chiba-u.jp; Phone:+81-43-226-2016; Fax:+81-43-226-2018

Ichiro Kawachi, Department of Social and Behavioral Sciences, Harvard School of Public Health, Boston, MA, United States of America, 677 Huntington Avenue, Boston, MA 02115
Email: ikawachi@hsph.harvard.edu; Phone:+1-617-432-3915

Appendix 1. Multivariate Poisson regression (A)RR and 95% CIs from MI analysis for support factors of depressive symptoms (n=3,340)

	Crude Model A1				Adjusted Model A2			
**Social support**	RR		95%CI		ARR		95%CI	
**Giving and receiving instrumental & emotional social support**								
**No emotional and no instrumetal social support**	1.00				1.00			
**one social support**	0.91		0.63	1.30	0.92		0.64	1.33
**two social supports**	0.83		0.63	1.09	0.82		0.62	1.09
**three social supports**	0.72	*	0.56	0.94	0.74	*	0.57	0.97
**four social supports**	0.48	**	0.38	0.61	0.53	**	0.42	0.68

MI: Multiple Imputation, RR: Rate Ratio, ARR: Adjusted Rate Ratio, CI: Confidence Interval
Model A2 adjusted for age, sex, living status (alone or not alone), equivalized income, all types of disaster damage [housing damage, loss of close relative(s), and loss of close friend(s)]
Four social supports:Giving and receiving instrumental & emotional support
Continuous variables: age and all types of disaster damage
Including indiividual whose GDS score was five or greater at baseline
*P-value for <0.05; **P-value for <0.01

Appendix 2. Distribution of GDS score at baseline survey among those who were included and excluded from the analysis

		Included sample	People who did not meet inclusion criteria	People who dropped out after baseline survey
GDS score at 2010	Mean (±SD)	1.6 (1.3)	7.4 (3.0)	5.6 (4.0)

Appendix 3. Poisson regression (A)RR and 95% CIs for support factors of depressive symptoms by each supporter (n=2,293)

	ARR	95%CI		ARR	95%CI		ARR	95%CI		ARR	95%CI	
**Before the disaster**												
	**Giving emotional social support to**			**Receiving emotional social support from**			**Giving instrumental social support to**			**Receiving instrumental social support from**		
**Spouse**	0.90	0.72	1.12	0.78 *	0.63	0.97	0.79 *	0.62	0.99	0.82	0.64	1.04
**Children living together**	1.00	0.78	1.27	1.05	0.83	1.32	0.92	0.73	1.17	1.13	0.92	1.39
**Children living apart / relatives**	1.03	0.84	1.27	1.01	0.82	1.24	0.88	0.70	1.10	1.00	0.81	1.23
**Neighbors**	1.23	0.99	1.52	1.19	0.93	1.51	0.85	0.54	1.34	0.75	0.41	1.37
**Friends**	0.80 *	0.65	0.99	0.85	0.69	1.05	0.77	0.51	1.17	1.01	0.67	1.53
**After the disaster**												
	**Giving emotional social support to**			**Receiving emotional social support from**			**Giving instrumental social support to**			**Receiving instrumental social support from**		
**Spouse**	0.73 **	0.58	0.91	0.62 **	0.50	0.77	0.67 **	0.54	0.83	0.62 **	0.49	0.78
**Children living together**	0.86	0.67	1.11	0.85	0.68	1.07	0.59 **	0.45	0.78	0.99	0.81	1.21
**Children living apart / relatives**	0.85	0.71	1.03	1.10	0.91	1.33	0.69 **	0.56	0.86	1.00	0.83	1.21
**Neighbors**	0.94	0.74	1.19	0.99	0.77	1.28	0.74	0.44	1.26	0.55	0.28	1.06
**Friends**	0.75 **	0.61	0.93	0.82	0.66	1.01	0.82	0.52	1.30	0.60	0.35	1.05

ARR: Adjusted Rate Ratio, CI: Confidence Interval: N/A: Not Applicable

Adjusted for age, sex, living status (alone or not alone), equivalized income, all types of disaster damage [housing damage, loss of close relative(s), and loss of close friend(s)]

**P*-value for <0.05; ***P*-value for <0.01
